# Standardization of PET/CT Performance Requirements for Whole-Body Quantitative Imaging: An International Proposal

**DOI:** 10.2967/jnumed.124.269349

**Published:** 2025-10

**Authors:** John J. Sunderland, Ronald Boellaard, John C. Dickson, Stephen A. Graves, Dale L. Bailey

**Affiliations:** 1 Department of Radiology, University of Iowa, Iowa City, Iowa;; 2 Department of Radiology and Nuclear Medicine, Amsterdam University Medical Centre, Amsterdam, The Netherlands;; 3 Department of Nuclear Medicine and Molecular Imaging, University Medical Center Groningen, Groningen, The Netherlands;; 4 Institute of Nuclear Medicine, University College London Hospitals Foundation Trust, London, United Kingdom; and; 5 Department of Nuclear Medicine, Royal North Shore Hospital, Sydney, New South Wales, Australia

**Keywords:** harmonization, standardization, scanner validation

## Abstract

Currently, PET scanner validation phantoms, methods, and acceptance criteria for clinical trials are not standardized. This situation generates substantial inefficiencies with many scanners being tested multiple times for different trials. Herein we propose a standardized PET scanner validation paradigm for clinical trials. **Methods:** At present, active PET scanner validation programs administered by the European Association of Nuclear Medicine Research Ltd. (EARL), the Society of Nuclear Medicine and Molecular Imaging Clinical Trials Network (CTN), and Australia New Zealand Society of Nuclear Medicine Australasian Radiopharmaceutical Trials Network are reviewed in detail to identify similarities, differences, strengths, and weaknesses. PET criteria that help define the quantitative performance characteristics most critical for clinical trials are identified. Historical quantitative scanner performance capabilities are reviewed, including increasing availability of primary and secondary standard activity measurements for calibration purposes. Methodologies for these phantom-based measurements are reviewed, and standardized approaches are recommended. **Results:** Phantom requirements, acquisitions, reconstruction, analysis, and acceptance criteria have all been developed to be reasonably aligned with current standard scanner validation approaches, while at the same time recommending improvements and clarifications where programmatic differences were identified. A scanner validation program based on the measurement of radionuclide specific scanner calibration and harmonized recovery coefficient performance is proposed. Quarterly calibration verification of ^18^F and annual calibration of other radionuclides are recommended. Accuracy of ±5% for ^18^F calibration and ±10% for other radionuclides are proposed acceptance criteria. Annual verification of EARL 2–concordant recovery coefficient performance using a National Electrical Manufacturers Association NU2 image quality phantom or CTN5 phantom imaged at an 8:1 target-to-background contrast is recommended, although contrast recovery coefficients, rather than recovery coefficients, are advised. **Conclusion:** An internationally standardized PET scanner validation paradigm is proposed. International adoption of such a system combined with a data-sharing system would create a more efficient, robust, uniform, and trustworthy scanner validation environment for clinical trials while improving clinical trial qualification efficiency, decreasing costs and mitigating duplication of testing.

The global growth and acceptance of clinical PET rests on numerous successful trials demonstrating both safety and efficacy of PET radiopharmaceuticals. Currently, there are nearly 20 PET radiopharmaceuticals with market authorization among the United States, Europe, and Australia, with many more diagnostic PET agents in both early- and late-stage clinical trials. The rapid growth of radiotheranostics is adding additional pressure to the PET drug development pipeline. This is expanding both the number of imaging sites engaged and the number of clinical trials in which individual imaging sites are participating.

In the context of both clinical practice and clinical trials, there are increasing use and dependence on quantitative metrics. The SUV is often used for clinical trial inclusion criteria or patient stratification in which both scanner calibration and recovery coefficient (RC) behavior are critical for generating comparable data across patient populations from different sites. Similarly, developing prognostic and predictive models for response to therapy is dependent on consistent quantitative behavior independent of the PET scanner used. These and other clinical use cases substantially depend on comparable and consistent data and PET image characteristics and quality across sites, which will only be optimized if defining and maintaining international standards can be agreed.

Validation of the proper functioning of equipment used in clinical trials is not only commonplace but often a requirement if the data will ultimately be used in support of a future regulatory application. In PET imaging, the scanner validation process typically uses 1 or more phantoms to assess the quantitative calibration of the scanner with the trial radioisotope(s) and may potentially also make additional measurements associated with image quality such as reconstructed spatial resolution–related metrics of the scanner. The scanner validation process is generally managed and documented by the sponsor of the clinical trial, the contract research organization (CRO) managing the trial or other qualified entities. Several international professional societies have independently created their own scanner validation infrastructures that are being used by both CROs and trial sponsors. Many CROs, independently, have developed their own validation methods and criteria.

Currently, scanner validation methods and acceptance criteria are neither standardized nor harmonized, resulting in inconsistent use of phantoms, an array of phantom fill concentrations and contrasts, differing acquisition protocols, and nonstandardized acceptance criteria, creating an uneven set of performance benchmarks. The current lack of standardization and harmonization is generating an unnecessary burden on clinical trial sites engaged in multiple clinical trials, in which individual scanners may be tested multiple times for different clinical trials while functionally gathering the same data using similar phantoms being judged by either similar or discrepant acceptance criteria. This lack of standardization is adding needless expense and complexity to study start-ups and unnecessary burden to busy clinics for minimal additional benefit.

Despite the different approaches, there appears to be a general convergence of international consensus on the metrics that should be tested to determine adequate scanner performance, general phantom geometries that will deliver necessary quantitative RC performance data, and acceptance criteria that demonstrate minimum performance requirements necessary to participate in clinical trials.

This convergence is presenting a significant opportunity for creation of an internationally standardized approach to PET scanner validation that would both simplify and make a more rigorous and consistent universal PET scanner validation foundation on which future clinical trials can rest.

It is important to emphasize that these PET performance recommendations are specific for clinical trial qualification. This purpose and application are substantially different from full National Electrical Manufacturers Association (NEMA) NU2 testing that is designed to characterize intrinsic PET scanner characteristics.

## GOALS

This article proposes an internationally standardized PET scanner validation program specifically for clinical trials, designed to achieve critical efficiencies, while maintaining necessarily high standards. The program is based on the combined experience of the authors, each of whom has contributed to the development of organized PET scanner validation programs in Europe, Australia, and the United States, combined with published (and unpublished) scanner performance data. The proposed validation framework has been designed to include, at a minimum, verification of radionuclide-specific quantitative calibration accuracy to within prescribed limits using a uniform phantom or other common phantoms meeting geometry requirements, and RC performance within prescribed limits (as a function of object size) using an asymmetric phantom approximating a human torso in size and shape, containing several spheres of different sizes.

The proposed paradigm attempts to strike a balance between not being overly prescriptive while providing sufficient guidance to assure reliably rigorous and comparable data across a range of scanner makes and models. Proposed acceptance criteria are designed to be consistent, to the extent possible, with published criteria defined by the European Association of Nuclear Medicine (EANM), EANM Research Ltd. (EARL), the Society of Nuclear Medicine and Molecular Imaging (SNMMI) Clinical Trials Network (CTN), and the Australia New Zealand Society of Nuclear Medicine (ANZSNM) Australasian Radiopharmaceutical Trials Network (ARTnet), although the proposed approach is neither formally approved nor sanctioned by the organizations. In fact, it is the organic convergence of scanner validation methodologies and acceptance criteria from each of the 3 organizations that has served as the inspiration for the paradigm proposed in this article.

**NOTEWORTHY**

- International PET/CT harmonization is essential for using PET as a quantitative imaging biomarker in clinical trials and research.
- International PET/CT harmonization leads to more efficient, robust, uniform, and trustworthy scanner validations for clinical trials.
- International PET/CT harmonization seems feasible because of the existing close alignment of procedures among 3 accreditation programs.

The envisioned end result of adoption of the proposed scanner validation paradigm would be an environment whereby a scanner validation procedure performed by a site for a particular scanner and validated as meeting standardized acceptance criteria by competent authority would be recognized uniformly across clinical trials as acceptable by CROs, trial sponsors, and regulatory authorities.

It is critical to understand that the minimum acceptance criteria as defined in the proposed validation framework are not meant to preclude a clinical trial sponsor or CRO from requiring additional acquisitions or reconstructions or more stringent acceptance criteria to assure that scanner performance is fit to inform trial end points.

A secondary goal is to develop methodologies for PET scanner validation that can be simply reengineered for similar processes in SPECT, thereby hopefully uniting the quantitative assessments of both modalities. However, quantitative SPECT performance measurement is less mature, and this goal is not yet ripe for inclusion in these current recommendations.

## METHODS

### PET Scanner Calibration

PET scanners are quantitative imaging devices. It is important to understand that voxel values in units of becquerel per milliliter are the native units of PET imaging systems, and that the SUV metrics commonly used clinically (SUV_mean_, SUV_max_, SUV_peak_) are calculated from these intrinsic becquerel per milliliter voxel values. In the context of these recommendations, we will be exclusively using becquerel per milliliter.

Quantitative PET scanner calibration procedures have been based on acquisitions of aqueous 20-cm diameter cylindric phantoms of known volume containing a known amount of radioactivity. Typically, this calibration is performed with ^18^F. Only since 2012 has it been possible for sites in the United States to routinely tie ^18^F measurements back to a U.S. National Institute of Standards and Technology (NIST)–traceable primary standard measurement made possible through availability of a commercial radionuclide calibrator source, which in turn has made it possible to tie PET scanner calibrations back to a primary NIST-traceable measurement. Some scanner manufacturers perform calibrations with 20-cm diameter ^68^Ge/^68^Ga epoxy phantoms of known volume and activity. However, the vendor-described calibration procedure requires the ^68^Ge activity concentrations to be recalibrated back to an aqueous ^18^F 20-cm cylindric phantom acquisition. This is because PET measured activity concentrations from phantoms with ^68^Ge activity in epoxy have inherent measurement bias due to attenuation properties of epoxy not being aligned with the human tissue on which CT-based PET attenuation corrections are based.

Integral to the PET scanner calibration accuracy is validation of radionuclide calibrator activity measurement for the radionuclide under study on which any accuracy assessment is ultimately based. (The term *radionuclide calibrator* is preferred to the more usual *dose calibrator* as the measurement performed is not of a *dose*, which can be ambiguous, but an amount of radioactivity.) An inaccurate radionuclide calibrator measurement of the sample used to fill the calibration-testing phantom will result in a baseline measurement difference (bias) proportional to the inaccuracy of the radionuclide calibrator measurement. Radionuclide-specific radionuclide calibrator verification is therefore a critical primary step in the PET scanner quantitative performance validation.

It is recognized that for SUV calculations if the same radionuclide calibrator is used for scanner calibration and patient activity measurement that measurement biases will cancel one another out. However, at many sites, multiple radionuclide calibrators are used, which creates opportunities for discordance. Also, PET scanners are typically calibrated for ^18^F, so a trial often using a different radionuclide that has a different measurement bias will also null this cancellation. Further, applications like dosimetry measurements for phase 1 and 2 clinical trials require absolute activity quantification, not SUVs. For these reasons, calibration of the radionuclide calibrator based on international standard primary or secondary standard measurements is recommended.

#### PET Scanner Calibration Validation for Clinical Trials

Validation of PET scanner calibration accuracy for clinical trials is a common phantom-based test used to assure clinical trial sponsors that the specific PET scanner is generating reliable quantitative data. It is standard procedure that a PET system designated for a clinical trial be tested for calibration accuracy for the specific radionuclide being used in the clinical trial.

#### Current Phantoms for PET Calibration Verification

Clinical trial scanner validation programs have routinely and successfully used the average of several background regions of the NEMA image quality (IQ) phantom to validate calibration accuracy (1). Both the EANM EARL PET oncology accreditation program and the ANZSNM ARTnet use the NEMA IQ phantom for this purpose. Similarly, the SNMMI CTN uses the background regions in the CTN3 or CTN5 anthropomorphic chest phantom to measure calibration accuracy. It is also common to test scanner calibration with a uniform cylindric phantom, such as the 20-cm uniform phantom that is typically used to calibrate the PET system.

Increasingly, longer-lived positron-emitting radionuclides are being incorporated into the development of PET radiopharmaceuticals. These include ^64^Cu (half life [*t*_1/2_] = 12.7 h), ^124^I (*t*_1/2_ = 4.2 d), or ^89^Zr (*t*_1/2_ = 3.25 d). After filling and imaging these phantoms, it is typical to store these phantoms for a minimum of 10 half-lives (more than a month for ^89^Zr and ^124^I). This makes it tempting to use smaller, less expensive phantoms with these longer-lived radionuclides for calibration measurements. ARTnet is currently using a custom-made 500-mL cylindric insert that fits in the NEMA IQ phantom’s central lung-insert space (2). SNMMI currently uses a smaller, inexpensive 1-L cylindric plastic bottle for these longer-lived radionuclide measurements.

#### Phantom Fill Procedures

Importantly, we recommend not using the manufacturer’s radionuclide calibrator default settings for measurement of activities for clinical trials unless no better alternative exists. Although radionuclide calibrator manufacturers are making efforts to make default calibration values accurate and tied to NIST measurements and other published data, significant radionuclide calibrator measurement biases of 5% to more than 20% have been recently reported and are not uncommon (3–7). In all cases, it is preferred to use a NIST (or similar) commercially available traceable radionuclide calibrator calibration source in the same measurement geometry as will be used for the phantom fill (and patient studies, ideally). Recommended approaches for volume measurement and activity measurement are described in detail in Supplemental Data sections 1.1 and 1.2 (supplemental materials are available at http://jnm.snmjournals.org). The scanner calibration measurement and verification are entirely dependent on knowing the precise and accurate activity in the phantom. Activities reported by a dose calibrator using default settings, or settings based on activities reported by suppliers, should be treated with skepticism unless tied to a primary or secondary measurement standard of the same device.

#### Activity Concentration Range, Scan Duration, and Axial Extent of Acquisition

Calibration validation should be an assessment of the ability of the PET system to accurately measure absolute concentration of radioactivity. In all cases, calibration validation should be performed with a fully independent phantom fill and acquisition, different from the phantom scan performed for the calibration. These procedures will be documented in the manufacturer’s recommended calibration procedures.

To ensure the best possible assessment is made, it is important that sufficient measured true coincidence photon events are acquired such that the accuracy of the measurement is not limited by statistical considerations. It is recommended that the radioactivity concentration used in the phantom be sufficiently high and acquisition duration sufficiently long to satisfy this requirement. Further, it is recommended that the phantom be scanned in its entirety, in a multibed fashion, such that with bed overlap all transaxial planes are of similar statistical quality.

For conventional PET scanners with 16–30-cm axial fields of view, radioactivity concentrations of approximately 3–7 kBq/mL are recommended with scan durations of at least 5 min per bed position. To achieve approximately equal sensitivity per transaxial slice, the acquisition should extend at least 5 cm past both ends of the phantom. It is important to note that having more than approximately 40 MBq of radioactivity in the scanner field of view (FOV) can begin to induce subtle pulse pileup and increasing dead time issues in some PET scanners that can impact the concentration measurement by 5% or more. This effect gets worse with increasing amounts of radioactivity.

#### Frequency of PET Scanner Calibration Validation

Currently, the frequency with which PET scanner calibration accuracy is checked for the purposes of a clinical trial is not standardized. Practical considerations play a role in this decision, given that many sites participate in multiple trials. Most commonly, an annual check of calibration is required, but more frequent measurements are sometimes requested. Many trials require a revalidation after major software updates, major service, after replacement of a ^68^Ge calibration phantom (as applicable), or after radionuclide calibrator service, which would impact the calibration of the combined radionuclide calibrator/scanner system pair. System recalibrations with an aqueous phantom are often performed quarterly as per manufacturers’ recommendations; this calibration will necessarily supplant the original calibration that was previously validated as accurate. Based on these facts, an argument for quarterly calibration revalidation could easily be made. To our knowledge, all PET manufacturers recommend validating the quantitative calibration performance after each recalibration using an independent phantom fill, acquisition, and clinical reconstruction. This suggests that quarterly quantitative calibration and verification are widely performed, and for many sites, this would require almost no additional work beyond the official recording of the procedure and uploading of the phantom scans for universal clinical trial validation purposes. It is recommended that both participating sites and scanner validation programs and CROs pay careful attention to the magnitude of changes in the scanner calibration factors during these quarterly checks. This could be a sensitive marker for an improper calibration update.

Given this background, it is recommended that if international PET scanner validation standards with data sharing were implemented then ^18^F (or ^68^Ga) calibration validation should be performed quarterly. For other less common radionuclides involved in clinical trials (e.g., ^64^Cu, ^89^Zr, ^124^I), given the added expense and the length of phantom quarantine during decay, annual verification is recommended.

#### Calibration Validation Analysis

Analysis of calibration phantom image data should sample uniform areas of the phantom throughout the FOV. For uniform phantoms, both EANM EARL and CTN follow similar approaches. The 2-dimensional circular regions of interest (ROIs) with a diameter of approximately 2 cm less than the diameter of the phantom (to avoid partial-volume effects) are drawn on contiguous transaxial slices. For each axial slice, the calibration accuracy (or bias in %) is derived as well as an average value across all slices. The calibration and uniformity for each slice as well as the global (slice averaged) value are displayed for inspection and reported. More details can be found in Kaalep et al. (8) as well as on the EARL website (https://earl.eanm.org/accreditation-specifications/) and the SNMMI Phantom Analysis Tool Web site (https://snmmi.org/Web/Clinical-Practice/Quality-and-Patient-Care/Phantom-Analysis-Toolkit-PAT.aspx). For the NEMA phantom or CTN phantoms, multiple ROI or volumes of interest (VOI) in uniform areas of the phantom are sampled and averaged.

For long–axial FOV PET systems (total-body PET), we recommend that the calibration phantom be scanned at 1/4, 1/2, and 3/4 of the axial FOV extent to generate evidence of uniform and accurate axial calibration along the extended FOV.

**FIGURE 1.**
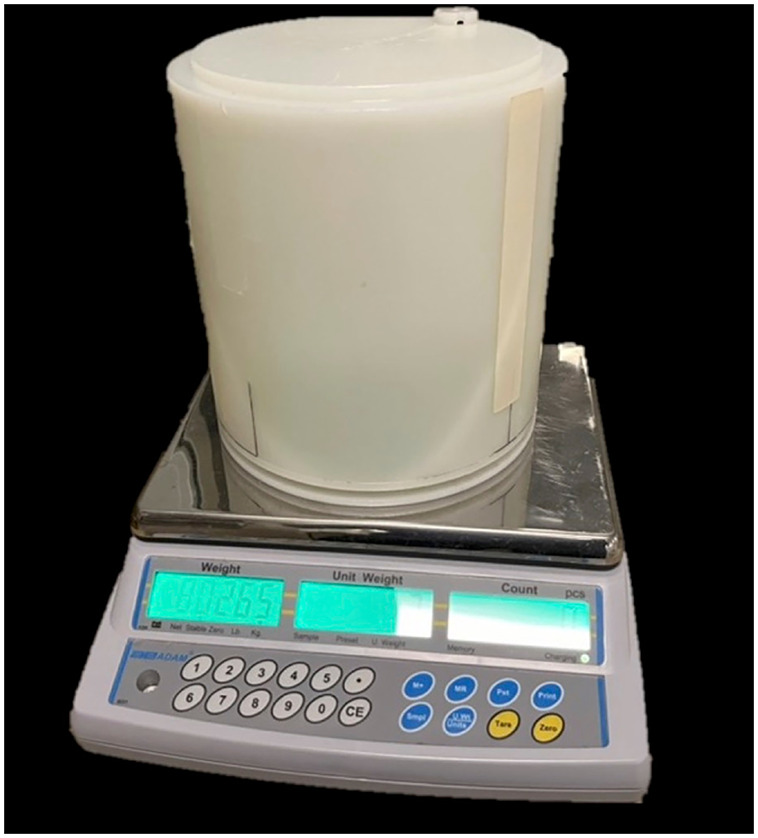
Photograph of 20-cm uniform cylinder phantom and digital scale. Digital scale is recommended for determining phantom fill volume.

Scanner calibration accuracy is reported as calibration bias, defined as

$$\mathrm{calibration}\;\mathrm{bias}=\frac{C_{\mathrm{Bkg}}}{A_{\mathrm{Bkg}}},$$ Eq. 1

where $C_{\mathrm{Bkg}}$ is the image-derived phantom concentration measurement as described above and $A_{\mathrm{Bkg}}$ is the activity concentration in the background as measured from a traceable radionuclide calibrator measured activity divided by the measured aqueous volume of the phantom (Fig. 1).

#### PET Scanner Calibration Acceptance Criteria

Calibration accuracy requirements for a clinical trial should be context- and end point–dependent. Historically, calibration accuracy within ±10% has been considered sufficient for validation of an appropriately calibrated PET system. However, PET imaging systems are innately capable of consistently better calibration accuracy. In ARTnet, and even for clinical use, PET/CT systems need to demonstrate calibration accuracy to within ±5% for ^18^F, as demonstrated from testing by formally accredited physicists (2). Sunderland et al. have reported monthly calibration accuracy of ±2% on 7 PET/CT systems over a period of 12 y when adhering to a standard NIST-based calibration testing regimen (9). EANM EARL and SNMMI CTN have both, historically, required ±10% accuracy with ^18^F and other radionuclides, as applicable for a specific clinical trial.

It is recognized that calibration accuracy may have differing levels of importance in different clinical trials. As such, we recommend a 2-tiered system for PET scanner qualification for clinical trials. Tier 1 is needed when quantitative measures are required. In the case when only visual assessment is performed, then tier 2 is proposed, but this will exclude future quantitative use of the data. For tier 1, it is recommended that demonstration of calibration accuracy of ±5% be maintained for ^18^F or ^68^Ga (given the ready availability of traceable primary standard measurements) through quarterly measurements and annual ±10% accuracy for other radionuclides. The decreased frequency and accuracy tolerance for other radionuclides recognizes the challenges associated with radionuclide costs and availability and storage and phantom availability, coupled with a lack of availability of radionuclide calibrator–traceable reference standards for some radionuclides. For tier 2, we propose that ±10% (annually) for all radionuclides is sufficient.

Phantoms used for calibration verification can provide several quantitative metrics that may be of use in informing clinical trials. The primary metric is calibration accuracy. However, meaningful information regarding noise metrics, reconstructed spatial resolution (10), and axial and radial uniformity can be meaningfully extracted from reconstructed datasets.

For the purposes of this international standardization proposal, we are only recommending reporting the calibration accuracy metric from the calibration measurement. However, this does not exclude the collection of other data and metrics and their potential use as acceptance criteria in a particular clinical trial where the context of a trial end point warrants such inclusion. In most cases, this additional information, if a trial chooses to require it, can be gleaned from the currently proposed phantoms and acquisitions.

A summarized list of scanner calibration validation recommendations is in Table 1.

**TABLE 1.** Summary Recommendations for PET Scanner Calibration Validation

Topic	Consensus	Comment
Acceptable phantoms	20-cm diameter cylinder	Other phantoms with sufficient uniform volume and regions that span axial extent of scanner ring allowable
	20-cm length minimum	
	Can be as small as 15-cm diameter	
	NEMA IQ phantom	
	CTN3 and CTN5 phantoms	
Volume measurement technique	Volume measured either by weight and density or through careful filling with calibrated flasks	
	Do not use manufacturer’s volume	
	Ideally use distilled water	
Radioactivity measurement radionuclide calibrator	Ideally radionuclide calibrator calibrated to NIST (or other) primary or secondary standard for specific radionuclide	Back-calibrating dose calibrator settings for non-^18^F radionuclides to well-calibrated PET system possible
	Activity measured in syringe before and after injection into phantom	
	Clocks synchronized with scanner clocks to <1 min	
Recommended activities	Approximately 3–7 kBq/mL recommended (0.08–0.19 μCi/mL) in uniform phantom	
Recommended acquisitions	Multibed acquisition extending at least 5 cm beyond end of phantom	Important to pay attention to zoom factor to assure totality of phantom in FOV; use manufacturer’s provided reconstructions; do not use research reconstruction tools
	>5 min/bed position	
Frequency of calibration validation	Quarterly using ^18^F and ^68^Ga	Validation dates begin on date of validation scan
	Other radionuclides annually	Revalidation of calibration for ^18^F or ^68^Ga should be performed after • Software update • Major service • Replacement of new ^68^Ge phantom calibration source (as applicable) • Radionuclide calibrator repair/maintenance
Quantitative metrics and acceptance criteria	Tier 1: quantitative end points • <±5% for ^18^F and ^68^Ga • <±10% for other radionuclides	
	Tier 2: nonquantitative end points or end points that are not strongly tied to quantitation • ±10% for all radionuclides	

## RECOMMENDATIONS

### PET Scanner RC Measurement

**FIGURE 2.**
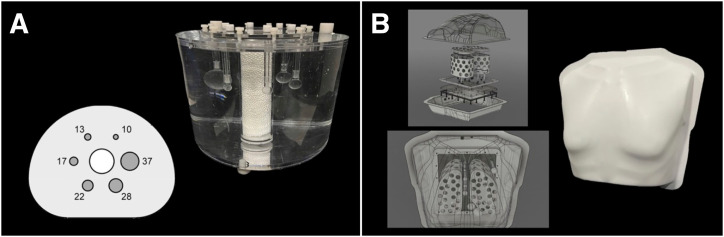
(A) NEMA NU2 IQ phantom. (B) SNMMI CTN5 phantom.

Several phantoms have been used to characterize the scanner contrast RC (CRC) performance over the past several decades. The NEMA IQ phantom has been the most common phantom used for this measurement, consisting of 6 spheres with diameters ranging from 10 to 37 mm spaced inside an approximately 10-L poly(methyl methacrylate) tank vaguely resembling the cross section of the human torso. It has a nonradioactive lung-equivalent cylindric insert in its center. This phantom has been used by EANM EARL since 2010 and by ANZSNM ARTnet since 2012. The NEMA IQ has been used in thousands of PET scanner validations and accreditations over that period. Since 2013, the SNMMI CTN has used an anthropomorphic chest-shaped phantom with lung inserts using 10–37-mm diameter spheres so the results can be unambiguously compared with NEMA IQ phantom results. The CTN phantom additionally contains a 7-mm diameter sphere to characterize RC performance and lesion detection for systems with higher spatial resolution. The CTN phantom has 2 approximately anthropomorphic-shaped low-density lungs for added imaging complexity (Fig. 2). Although less common than the NEMA IQ phantom, the CTN phantom is commonly used in the PET validation space that is used in over 1,000 clinical trial PET scanner validations over the last decade.

Several RC characterization paradigms have been developed and are in routine use. The standard NEMA methodology reports CRCs using the NEMA IQ phantom. The CRC curve characterizes a PET system’s ability to measure activity concentrations of objects impacted by the limited spatial resolution of PET images under variable contrast conditions; it functionally quantifies the impact of partial-volume effects as a function of object size at a given contrast for the *i*th sphere with the formula

$${\mathrm{CRC}}_{i}=\;\frac{\left(\frac{C_{\mathrm{sphere}(i)}-\;C_{\mathrm{Bkg}}}{C_{\mathrm{Bkg}}}\right)}{\left(\frac{A_{\mathrm{sphere}\;}-\;A_{\mathrm{Bkg}}}{A_{\mathrm{Bkg}}}\right)},$$ Eq. 2

where *C*_sphere(_*_i_*_)_ is the PET scanner measured activity concentration (Bq/cm^3^) of each of the *i*th spherical inserts, derived from an ROI or VOI of each sphere. *C*_Bkg_ is the PET scanner measured activity concentration in the background of the phantom. *A*_sphere_ is the known activity concentration in the sphere as measured from a radionuclide calibrator measured activity divided by the known aqueous volume of the dilution flask, and *A*_Bkg_ is the activity concentration in the background as measured from a radionuclide calibrator measured activity divided by the known aqueous volume of the phantom.

It is important to note that the CRC value is not impacted by the calibration bias of the scanner calibration. Both *C*_sphere(_*_i_*_)_ and *C*_Bkg_ are scaled up or down by the calibration bias proportionally in the numerator of Equation 2, and the effect cancels out.

The simpler RC metric is calculated without regard to contrast and is calculated using the same definitions as above for each sphere as

$${\mathrm{RC}}_{i}=\frac{C_{\mathrm{sphere}(i)}}{A_{\mathrm{sphere}}}.$$ Eq. 3

It is important to note that, although simpler to calculate, for radionuclides apart from ^18^F, the RC metric may be linearly affected by the calibration bias. That is, the measured *C*_sphere(_*_i_*_)_ is linearly scaled by the calibration bias, whereas *A*_sphere_ is not. This results in the calculated RC curve moving up or down, depending on the measured calibration bias of the scanner. If the same radionuclide calibrator is used for calibration of the scanner and phantom and patient activities, and the radionuclide is ^18^F (the same radionuclide the scanner was calibrated with), then this effect cancels out. This situation is not always the case. In the case of non-^18^F radionuclides, since the scanner is calibrated for ^18^F activities, the potential for the above biases still exists, giving potential for unrelated bias for the non-^18^F radionuclide calibrator activity measurement.

Many variants of the CRC curve can be generated for a single PET scanner system. A CRC (or RC) curve can be generated using 2-dimensional circular ROIs or 3-dimensional spheric VOIs with the precise size of the spheric inserts, and the average concentration can be calculated within each. This approach mimics the NEMA NU 2 image quality formalism, although its practical clinical use is not recommended given that we often do not know the precise size and shape of a target tissue in a patient. Similarly, a CRC (or RC) curve can be generated from the identical ROIs or VOIs and by recording the maximum single voxel concentration in each sphere. This is commonly performed both clinically (i.e., SUV_max_) and in the context of PET scanner validations. However, noise associated with maximum pixel values tends to result in noisier CRC (or RC) values and, in general, biases the curves higher. This drawback can be mitigated by collecting high statistic scans for purposes of CRC (or RC) measurements. Alternatively, a 1-cm^3^ spheric VOI can be moved around within a spheric VOI to identify the highest 1-cm^3^ average concentration (the peak concentration for each sized sphere) (11). The usefulness of peak-based measurements diminishes as object sizes decreases—for 13-mm diameter spheres and smaller, the peak measurement will include background voxels in the calculation.

It is also important to note that a different CRC curve for any given scanner will be generated with different sphere-to-background ratios because of spill-in and spill-out effects. Relative contrast between the spheres and the background affects recovery, with the effect of contrast on the CRC (or RC) curve diminishing as the contrast increases. Lastly, different reconstruction parameter settings (iterations, subsets, and postreconstruction filter) will dramatically impact the shape and bias of the CRC (or RC) curve for each scanner.

Currently, most scanner manufacturers provide a broad array of image reconstruction options to users so that they can adapt their reconstructions to the clinical task at hand and the preference of the reading physicians. This results in significant challenges to entities charged with characterizing, harmonizing, and validating PET scanner RC performance for a clinical trial.

To manage this potential quantitative performance range in the context of clinical trials, several reconstruction harmonization initiatives have been launched with the intent of limiting the range of allowed CRC (or RC) performance. Adjusting the curve to meet the requirements of a prescribed harmonized RC range is practically achieved by increasing or decreasing the number of iterative updates (defined as iterations × subsets) and applying postreconstruction 3-dimensional gaussian smoothing filters with different widths. These methods are discussed in more detail in Supplemental Data section 1.3.

Further complicating this space are newer PET radiopharmaceuticals labeled with ^89^Zr, ^64^Cu, or ^124^I. Given their relatively long half-lives, low positron branching ratios, and circulation times on the order of tens of hours to days, significantly lower injected activities are used because of radiation-absorbed dose considerations. The combined result of these factors leads to PET images with a factor of 10 or more fewer photons, resulting in demonstrably noisier images given the same reconstruction parameters. The resulting noise will bias maximum voxel-based measurements, such as SUV_max_, higher.

#### RC Measurement for Clinical Trials

Measurement of RC performance has been another standard practice in PET scanner clinical trial qualification. For highest utility within the context of clinical trials, RC measurement as a function of object size is best standardized in methodology with performance harmonized across scanners.

#### PET Scanner RC Performance Validation

RC evaluation for the purpose of qualification of a PET scanner for clinical trials should be consistent with the intent of the trial and any quantitative end points. In general, EARL, CTN, and ARTnet attempt to generate some level of reconstruction harmonization among all scanners in the trial. The idea is to force all scanners in the trial into a similar range of RC behavior through prescriptive reconstructions that will generate similar quantitative results, directly comparable to one another. There are currently some differences in the way EARL, CTN, and ARTnet handle RC measurements.

EARL has 2 different performance ranges, EARL 1 and EARL 2 criteria (8,12). The higher performance EARL 2 criterion was implemented in 2019 to take advantage of the higher spatial resolution and performance of newer PET technologies. Ongoing clinical trials originally using EARL 1 continue to use this specification; all newer trials have migrated to EARL 2. CTN shares a common lower boundary for CRC behavior with EARL 1. CTN has not yet migrated to EARL 2 specifications because of challenges of some older systems meeting the criteria. CTN does not currently have a static upper range boundary that is applicable to all trials. For ^18^F and ^68^Ga applications, upper boundaries closely align with EARL 1. However, their scanner validation process philosophically requires that the RC phantom data are collected in a manner similar to research subject image data specific to the trial. For clinical trials with longer-lived radionuclides, clinical trial images (and therefore phantom images) will be count-deficient because of limited injected activity and low positron branching ratios. This will result in noise-driven SUV_max_ values typically higher than those seen in statistically robust PET images. ARTnet have implemented calculations that are specified for the NEMA IQ phantom in NEMA NU 2-2018 (13), including CRC and background variability, in addition to quantitative radioactivity concentration measurements in spheres and background compartments and the corresponding SUV_max_ and SUV_mean_.

EARL, CTN, and ARTnet also currently diverge from one another regarding phantom contrast. EARL uses a sphere-to-contrast ratio of close to 10:1 with the NEMA IQ phantom. ARTnet protocols require an 8:1 contrast ratio with the NEMA IQ phantom. CTN uses a radiopharmaceutical-specific contrast ratio based on radiopharmaceutical-specific data provided by the sponsor. This leads to trial-specific phantom fills, with trial-specific contrasts ranging from as low as 4:1 to as high as 20:1. The different contrasts lead to different CRC curves because CRC explicitly includes contrast in its definition.

EARL uses a background concentration of approximately 2 kBq/mL at the start of NEMA IQ phantom measurement (approximately 20 MBq) to simulate background in an patient receiving FDG with an average radioactivity administration. ARTnet suggest using an amount of radioactivity equivalent to 50–100 MBq (5–10 kBq/mL) of ^18^F in the background or, for lower amounts of radioactivity or positron branching ratio, to extend the acquisition time. CTN is more routinely engaged in clinical trials with more novel agents and tune their phantom fill to match relevant background concentrations of the trial radiopharmaceutical.

There is also a difference in reporting of contrast recovery metrics. EARL reports their curve in terms of simple RC*_i_* as in Equation 3. ARTnet and CTN reports CRC*_i_*. There are challenges associated with each of these approaches, particularly as it relates to definition and alignment with harmonization criteria.

The limitation of the EARL approach is that the RC*_i_* curves are affected linearly with calibration bias, which potentially impacts meeting the validation criteria.

CTN and ARTnet report CRC*_i_*. Importantly, CRC*_i_* intrinsically normalizes for calibration bias. This does not present a challenge for comparison with RC*_i_* values as they can be uniquely calculated from one to the other with

$${\mathrm{CRC}}_{i}=\frac{\left(\frac{{\mathrm{RC}}_{i}\;\times \;\mathrm{contrast}}{\mathrm{calibration}\;\mathrm{bias}}\;-1\right)}{\left(\mathrm{contrast}-1\right)}.$$ Eq. 4

**FIGURE 3.**
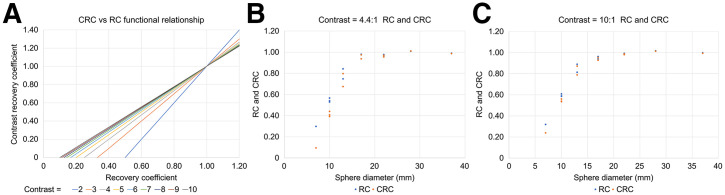
(A) Functional relationship between RC*_i_* and CRC*_i_* from Equation 4. (B) Actual measured and calculated RC and CRC phantom data from CTN5 phantom at 4.4:1 contrast. (C) Actual measured and calculated RC and CRC phantom data from CTN5 phantom at 10:1. Reconstructions used for these studies were tuned to EARL 1 and CTN criteria and not more aggressive EARL 2 criteria.

The challenge with using CRC*_i_* within a harmonization paradigm is that the CRC*_i_* curve shifts with contrast, and technically, this would require different harmonized target curves for each contrast (Fig. 3). This deficiency is avoided if the target-to-background ratio is standardized.

#### Recommended Phantoms for RC Verification

Historically, RCs have been reported for hot spheric objects of different sizes in a warm background. Both the NEMA IQ phantom and CTN phantoms have fillable spheric objects and use the analysis of their resulting CRC*_i_* curve as the basis for their performance validation. Sphere sizes are standardized to include spheres with diameters of 10, 13, 17, 22, 28, and 37 mm. The CTN phantom contains an additional 7-mm diameter sphere. Both phantoms have noncylindric phantom geometries with volumes of more than 9.0 L that approximate the shape and size of a human torso and have attenuation and scatter challenges in the form of additional lung-density inserts.

Recommendations in this document specifically attempt to avoid stifling innovation in the phantom development space. Nonetheless, with the explicit intention of developing comparable data across scanner validation programs, some guidelines for acceptable phantoms and phantom design are necessary.

Specific recommendations are summarized in Table 2 and are discussed below. A noncylindric design vaguely simulating a human torso in both size and shape, with a volume in excess of 9 L and an axial length of greater than 18 cm, is suggested. The phantom inserts should include at least 1 significant nonunit density insert to challenge both attenuation and scatter correction. At least 5 fillable spheric inserts with wall thicknesses of approximately 1 mm or less spanning the range of inner diameters of 10–28 mm at minimum is required. Adding additional spheres beyond this mandated sphere diameter range is both allowed and encouraged, as PET imaging technology and achievable resolutions are no longer significantly challenged by current NEMA IQ sphere sizes. Standard phantoms with smaller spheres are likely to emerge soon. Extension of the harmonized CRC curve down to smaller sphere sizes will be implemented when sufficient data are generated.

**TABLE 2.** Summary Recommendations for PET Scanner RC Performance

Major topic	Consensus	Comments
Acceptable phantoms	>9 L	
	Noncylindrical; ideally torso shaped	
	NEMA-sized spheres, at minimum	
	At least 18 cm in length	
Frequency of validation	Annually	
Volume measurement technique	Volume measured either by weight/density or through careful filling with calibrated flasks	
	Do not use manufacturer’s volume	
	Ideally use distilled water	
Radioactivity measurement of radionuclide calibrator	Ideally radionuclide calibrator calibrated to NIST (or other) primary standard for the specific radionuclide	
	Activity measured in syringe before and after injection into phantom	
	Clocks synchronized with scanner clocks to <1 min	
Recommended concentrations/contrast	Background concentration targeting 2–6 kBq/mL at scan time	Allows for additional shortened acquisition (or shortened replay of original data) of same phantom fill to simulate lower concentrations and total counts, if considered necessary
	Sphere to background contrast 8:1	
Recommended scan duration and acquisitions	Acquisition length should be a multiple bed position scan extending at least 5 cm from each end	Acquisitions should archive both reconstructed image data and list mode data
	Acquisition time of 5 min per bed position is required	Additional longer acquisitions, per clinical trial allowed
	Phantom centered in FOV	Rebinning of acquisition data to shorter scan times allowed
Recommended reconstructions	≥192 × 192 matrix	Additional reconstructions, per clinical trial allowed
	*X* and *Y* voxel dimensions: minimum, 1.5 mm; maximum, 2.75 mm	
	reconstruction targeting the center of the CRC acceptance criteria range in Table 3	

Precise and accurate measurements of both the phantom volume and activity are essential for the background measurement of activity concentration. Similarly, measurement of the activity concentration of the solution put into the spheres is of equal importance for measurement of CRC*_i_*. Recommended approaches for activity and volume determination are described in detail in Supplemental Data sections 1.4 and 1.5. Additionally, details on methodologies to assure accurate contrast is achieved are described in Supplemental Data section 1.6.

#### RC Validation Summary Recommendations

Regarding the axial extent of the phantom acquisition and to provide roughly equal sensitivity across the phantom, it is recommended that the phantom acquisition use a multibed position acquisition that extends at least 5 cm beyond either end of the phantom. Continuous bed motion is permitted.

To enhance comparability of PET scanner quantitative performance as measured by the CRC*_i_* (or RC*_i_*) curve, collecting good statistical quality image data is a high priority. To achieve this, it is recommended that the background activity in the phantom be at least 2 kBq/mL of ^18^F at the time of imaging, with acquisition times of least 5 min per bed position (or equivalent). The background concentration should not exceed 6 kBq/mL. Correction for dead time on some PET systems may be challenged at a concentration of 6 kBq/mL, and measured background concentrations may begin to manifest measurable concentration bias. ^18^F, with its low-energy positron energy and hence shorter mean free pathlength, will generate a best-case CRC*_i_* (or RC*_i_*) curve, which is generally recommended for clinical trial validation. It is recommended that the acquisition be collected in list mode, if available, and that the list-mode data be archived along with the images.

Given the shorter half-life and relatively ubiquitous availability of ^68^Ga for sites participating in ^68^Ga clinical trials, it is suggested, but not required, that ^68^Ga be used for CRC*_i_* (or RC*_i_*) curve generation for clinical trials using ^68^Ga. The higher-energy positron from ^68^Ga will result in small but measurable CRC*_i_* differences from ^18^F. If a qualifying ^18^F phantom acquisition exists and is still valid, it will suffice for the CRC measurement.

Sphere-to-background contrast is recommended to be 8:1 to be consistent with ARTnet and close to the EARL 10:1 contrast. This results in approximately 16 kBq/mL sphere concentrations at imaging time for a 2-kBq/mL background fill. Sphere concentrations would be linearly scaled for higher background concentrations. Figure 3 demonstrates that the CRC*_i_* and RC relationship converges asymptotically to a relatively consistent slope at contrasts of 8:1 and above (including the 10:1 EARL contrast), making CRC*_i_* largely invariant to small phantom fill inaccuracies. Summary recommendations for RC phantoms and data collection are presented in Table 2.

#### Reconstruction Considerations

Quantitatively harmonized reconstruction methodologies are considered central to this PET scanner validation paradigm. To be forward-looking, and so as not to degrade available performance of most currently active PET systems that have substantially higher performance than previous generation scanners, it is prudent to move, universally, to EARL 2 criteria. The empirically developed range is designed to accommodate both lightly filtered standard iterative reconstructions and point response function reconstructions, typically with more gaussian filtration. Recommended upper and lower maximum voxel-based CRC*_i_* limits based on published EARL 2 RC*_i_* at the proposed 8:1 contrast are listed in Table 3. Scanner model–specific reconstruction protocols tested to meet the proposed EARL 2 criteria should be used but are beyond the scope of these recommendations. It should be noted that the lower acceptable range somewhat unexpectedly is above the 1.0 CRC level for larger spheres. This is empirically driven by a combination of single voxel noise expected with clinical statistics and Gibbs artifacts associated with point response function reconstructions.

**TABLE 3.** Recommended PET Scanner Maximum Voxel-Based CRC Acceptance Criteria (Contrast = 8)

Diameter (mm)	Minimum CRC	Maximum CRC
10	0.45	0.86
13	0.83	1.25
17	1.00	1.43
22	1.01	1.37
28	1.01	1.30
37	1.06	1.33

**FIGURE 4.**
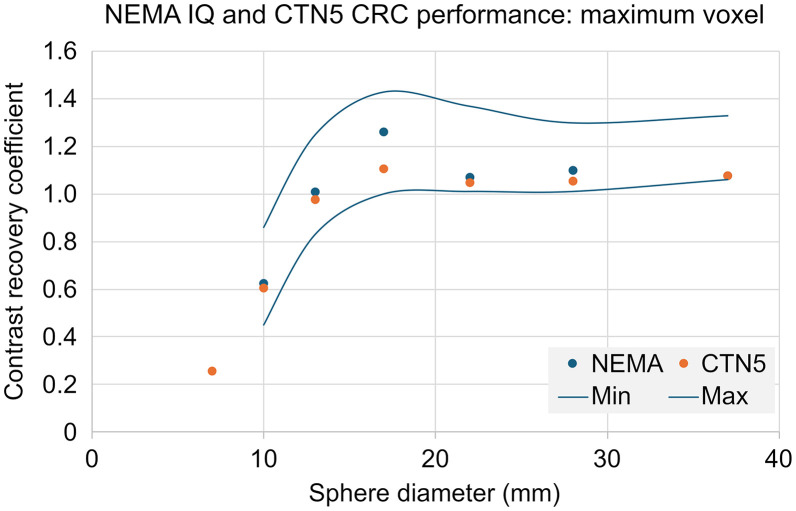
NEMA IQ and CTN5 phantom study on single PET/CT system using identical specified fill concentrations (4 kBq/mL), contrast (8:1), with proposed CRC*_i_* acceptance criteria. Reconstructions performed with point response function reconstruction with 24 iterative updates and 5-mm gaussian filter. Note that, despite identical concentrations and reconstructions, significant Gibbs artifact–driven difference appears in 17-mm sphere.

As the proposed test paradigm is restricting the contrast to a consistent 8:1 ratio, we recommend moving universally to a CRC*_i_* paradigm (as opposed to an RC*_i_* paradigm). We suggest this for 2 primary reasons. First, it automatically makes the curve insensitive to calibration bias. Second, it is consistent with NEMA methodologies (Fig. 4).

#### Frequency of PET Scanner RC Validation

Annual validation of CRC harmonization is proposed.

#### CRC Calculation Validation Analysis Approaches

VOIs that use the physical size of the sphere’s inner diameters are recommended for calculation of CRCs. Current proposed acceptance criteria are based solely on maximum CRC. Calculations of maximum, mean, and peak CRC measurements are recommended to be collected and archived in the event validation program modifications for consideration in the future. Using the centroid of the PET-measured sphere for centering a VOI of the precise size of the sphere is a recommended approach for VOI generation. Using CT images is not recommended, as coregistration errors of up to 2 mm are common and can impact VOI measurements of mean concentrations. Using a thresholding method for VOI definition is possible, but this obviates the requirement of a consistent mean VOI measurement across scanners. Maximum and peak concentration values are largely insensitive to VOI definition.

It is recommended that automated software packages that calculate CRC*_i_* values for purposes of scanner validation be tested against one another to understand biases and differences that may exist and to facilitate changes in code necessary to achieve confidence in comparability of results. One potential factor that may contribute to differences, even within maximum value measurements, is related to reconstructed voxel size and whether the software supersamples the image data before calculation. Differences in methodology relative to this calculational approach will need to be considered when comparing software solutions for use in the proposed harmonized paradigm.

#### Optional Acquisitions, Fills, and Reconstruction Variants

For those situations where lower count scans, different contrasts, or additional reconstructions are deemed to have value to inform a clinical trial, several simple additional procedures are described in Supplemental Data section 1.7.

#### Final Validation Decisions

Final scanner validation decisions in the envisioned internationally standardized paradigm would be at the discretion of the validating authority for the clinical trial based on scanner phantom quantitative performance data and data available through a shared database resource.

It is important to remember that the primary goal of the proposed PET scanner validation program is to ensure a properly performing and calibrated PET system capable of reliable and accurate data for clinical trials. The tests and performance standards aim to be simple to perform and quantitatively rigorous. However important the quantification may be, having trained human eyes to view the phantom images is also recommended. This is necessary to identify unexpected image artifacts that may not manifest in the limited quantitative metrics collected, such as Gibbs artifacts, when point-spread function corrections are used. There may be cases where, for example, a sphere (or spheres) may be only partially filled, resulting in an RC curve with 1 (or more) points outside of harmonized acceptance range, but the trajectory of the CRC curve is clearly within boundaries and the outliers explainable. Discretion from validation authorities should be permitted, assuming trained personnel are engaged.

Qualifications of personnel responsible for performing review and analysis of PET scanner validation submissions need to be defined by their managing authority. In all cases, however, reviewers should have appropriate education and training in PET image analysis. It is envisioned that reviewers will be trained nuclear medicine technologists or physicists, but other backgrounds are possible. However, because sometimes subtle but important scanner performance issues arise, a PET-experienced physicist should be available for consultation.

Reviewer responsibilities should include, but are not limited to, verifying that the phantom fill(s) and scan acquisitions were performed in accordance with protocols; verifying that reconstructions performed and submitted meet the expected criteria (reconstruction algorithm, image matrix size, voxel size); determining that the specified quantitative criteria for acceptable performance are achieved for each defined criterion; ensuring that the phantom scans are free from unexpected artifacts; assessing PET and CT coregistration; and providing a final decision regarding whether the scanner passes the validation.

## DISCUSSION

The proposed standardized scanner validation paradigm has been constructed as a middle-ground between existing scanner validation paradigms, not only by professional societies but also by the authors’ experience with CROs and their scanner validation programs that often closely parallel the proposed paradigm.

If these recommendations are adopted universally, it would result in several tangible benefits to trial sponsors, CROs, participating trial sites, and regulatory bodies. These benefits include, but are not limited to, providing substantial efficiencies for trial sponsors and CROs in defining the scanner validation protocols in their imaging manuals; demonstrating that scanner calibration validation can be applied for all clinical trials with the radionuclide for the duration defined by the standards (3 mo for ^18^F or ^68^Ga, and 1 y for other radionuclides); demonstrating that RC validation of a scanner for a single trial will apply for all clinical trials for the duration defined by the standards (1 y); scheduling that will result in generally fewer phantom scans for a clinical trial; reducing the cost for the trials by requiring fewer scans; establishing uniform performance requirements for all PET systems in the clinical trial space; facilitating more comparable and reproducible quantitative PET imaging data for the trials; providing more rapid site validation; and providing a validated testing strategy to remove this burden from the sponsor, who is sometimes not able to provide appropriate guidance regarding the degree of testing required.

Currently, the proposed validation program remains silent on acceptance criteria associated with image noise, axial or radial uniformity, and lesion detectability. Individual trials are free to add reporting and acceptance criteria associated with these parameters, but they currently are not part of the envisioned program.

It is acknowledged that this field is not static, and developed technologies will impact the proposed standards in the near future. However, this proposal is a collection of structured recommendations built into a construct. As the field evolves, specific recommendations can replace or supplement existing ones. For example, a NEMA IQ phantom prototype is being tested with spheres as small as 6 mm. Adoption would necessarily require redefining acceptable phantom design and testing and extending acceptable CRC*_i_* criteria for smaller spheres. It would require a reworking of the CTN5 phantom to accommodate smaller spheres. Innovative phantoms meeting newly defined criteria would be acceptable, but having a standard construct on which to make focused changes makes the process of change easier, more controlled, and more likely to happen.

## CONCLUSION

The universally standardized PET scanner validation program described herein has been devised to enhance the rigor associated with PET scanner validations for clinical trials while at the same time making the process more globally efficient and creating a uniform performance playing field. Phantom requirements, acquisitions, reconstruction, analysis, and acceptance criteria have all been developed to be reasonably aligned with current standard approaches, while at the same time improvements and clarifications in which programmatic differences were identified were recommended. Proposed methodologies attempt to avoid being overly prescriptive while maintaining consistent and meaningful PET/CT performance requirements for metrics that will help assure robust and comparable quantitative data across international clinical trials.

## DISCLOSURE

John Sunderland is a member and past chair of the SNMMI CTN Scanner Validation Committee (unpaid). Ronald Boellaard has received a research grant from Siemens Healthineers (not related to this paper) and is (unpaid) scientific chair of the PET accreditation program of EARL. John Dickson is (unpaid) scientific chair of the SPECT accreditation program of EARL. No other potential conflict of interest relevant to this article was reported.
